# Association of Preterm Birth With Myocardial Fibrosis and Diastolic Dysfunction in Young Adulthood

**DOI:** 10.1016/j.jacc.2021.05.053

**Published:** 2021-08-17

**Authors:** Adam J. Lewandowski, Betty Raman, Mariane Bertagnolli, Afifah Mohamed, Wilby Williamson, Joana Leal Pelado, Angus McCance, Winok Lapidaire, Stefan Neubauer, Paul Leeson

**Affiliations:** a Oxford Cardiovascular Clinical Research Facility, University of Oxford, Oxford, United Kingdom; b Oxford Centre for Clinical Magnetic Resonance Research, Division of Cardiovascular Medicine, Radcliffe Department of Medicine, University of Oxford, Oxford, United Kingdom; c Oxford University Hospitals NHS Foundation Trust, Oxford, United Kingdom; d Hôpital du Sacré-Cœur de Montréal Research Center (CIUSSS Nord-de-l’Île-de-Montréal), School of Physical and Occupational Therapy, McGill University, Montréal, Quebec, Canada; e Department of Diagnostic Imaging & Applied Health Sciences, Faculty of Health Sciences, Universiti Kebangsaan Malaysia, Kuala Lumpur, Malaysia

**Keywords:** diastolic function, diffuse fibrosis, extracellular matrix, myocardial fibrosis, preterm birth, CI, confidence interval, CMR, cardiovascular magnetic resonance, ECV, extracellular volume fraction, LGE, late gadolinium enhancement, LV, left ventricular

## Abstract

**Background:**

Preterm birth affects about 10% of live births worldwide and is associated with cardiac alterations. Animal models of preterm birth suggest that left ventricular functional impairment may be due to an up-regulation of myocardial fibrosis.

**Objectives:**

The aim of this study was to determine whether diffuse left ventricular fibrosis is evident in young adults born preterm.

**Methods:**

One hundred one normotensive young adults born preterm (n = 47, mean gestational age 32.8 ± 3.2 weeks) and term (n = 54) were included from YACHT (Young Adult Cardiovascular Health sTudy). Left ventricular structure and function were quantified by cardiovascular magnetic resonance and echocardiography. Intravenous administration of a gadolinium-based contrast agent during cardiovascular magnetic resonance was used to quantify focal myocardial fibrosis on the basis of late gadolinium enhancement and, in combination with T1 mapping, to quantify diffuse myocardial fibrosis on the basis of assessment of myocardial extracellular volume fraction.

**Results:**

Adults born preterm had smaller left ventricular end-diastolic and stroke volumes, with greater left ventricular mass and wall thickness (*P* < 0.001). In addition, longitudinal peak systolic strain and diastolic strain rate by both cardiovascular magnetic resonance and echocardiography, and E/A ratio measured by echocardiography, were lower in preterm-born compared to term-born adults (*P* < 0.05). Extracellular volume fraction was greater in preterm-born compared with term-born adults (27.81% ± 1.69% vs 25.48% ± 1.41%; *P* < 0.001) and was a significant mediator in the relationship between gestational age and both longitudinal peak diastolic strain rate and E/A ratio.

**Conclusions:**

Preterm-born young adults have greater extracellular volume fraction in the left ventricle that is inversely related with gestational age and may underlie their diastolic functional impairments.

Preterm birth (<37 gestational weeks) affects >10% of live births worldwide (1) and abruptly interrupts the trajectory of cardiac development, leading to neonatal (2) and long-term (3) morbidity. Large-scale birth registries investigating the impact of being born preterm have shown an increased risk for cardiovascular diseases by childhood and adulthood, including heart failure (4,5), ischemic heart disease (6), and early cardiovascular-related mortality (7).

In line with an increased cardiovascular risk, left ventricular (LV) morphologic and functional changes in people born preterm are first observed in the neonatal period and continue into infancy, childhood, and young adulthood (8). These potentially adverse alterations extend across gestational age categories of prematurity and include smaller internal cavity dimensions and volumes, reduced systolic and diastolic function, and an increased rate of hypertrophy from childhood to adulthood (8), though there is variability in LV mass findings across studies (9,10). This is consistent with findings from animal models of prematurity, which show smaller cardiac dimensions and greater myocardial interstitial fibrosis (11,12). Of note, reduced LV systolic and diastolic function measured using echocardiography in a preterm rat model relate to an up-regulation of cardiac fibrotic pathways (13,14).

To date, there have been no human studies investigating myocardial fibrosis in adults born preterm and how this relates to changes in systolic and diastolic function. Cardiovascular magnetic resonance (CMR) imaging offers the opportunity to assess focal myocardial fibrosis with late gadolinium enhancement (LGE) and diffuse myocardial fibrosis by pre- and postcontrast T1 mapping to measure myocardial extracellular volume fraction (ECV), without requiring direct access to tissue (15,16). We have therefore used CMR and echocardiography to determine whether preterm-born adults have greater LV myocardial fibrosis and whether this relates to the degree of prematurity and their known reductions in systolic and diastolic function.

## Methods

### Study population

Subjects were recruited into YACHT (Young Adult Cardiovascular Health sTudy; NCT02103231), a cross-sectional, case-control study of preterm-born (<37 gestational weeks) and term-born (≥37 gestational weeks) young adults (17,18). Recruitment methods included open recruitment in the local Oxford community using posters and e-mails, mailed invitations from the John Radcliffe Hospital birth registries, patient invitation through the John Radcliffe Hospital specialist hypertension clinic, and invitations to previous study participants who had indicated interest in future study participation. Inclusion and exclusion criteria for this analysis were age 18 to 40 years, body mass index <40 kg/m^2^, availability of detailed birth history information, no current or history of hypertension treatment, no contraindications to CMR or gadolinium-based contrast agent, no documented cardiac or cerebrovascular disease, and awake ambulatory blood pressure <135/85 mm Hg. Birth and family history data were attained via access to medical notes and by questionnaire. All research studies took place at the Oxford Cardiovascular Clinical Research Facility and Oxford Centre for Clinical Magnetic Resonance Research, where subjects underwent anthropometric, demographic, and cardiovascular phenotyping. All data were coded with subject- and study-specific identifications (eg, YT-001) to ensure anonymity and blinded analysis. YACHT was granted ethical approval by the South Central Berkshire Research Ethics Committee (14/SC/0275) in the United Kingdom.

### Study visit

#### Anthropometry, blood pressure, and blood samples

Before attending their study visit, all participants fasted for 12 hours, consuming only water during this time to keep measurement conditions similar between participants. Trained clinical research investigators completed all measurements. As previously described (17,18), height was measured to the nearest centimeter and weight to the nearest 0.1 kg using an integrated height and weight measurement station (Seca). Twenty-four-hour ambulatory blood pressure monitoring was initiated at the end of the study visit using oscillometric, ambulatory devices (TM-2430, A&D Instruments). Venous blood samples were collected and full blood count was measured on the day of the study visit, with additional samples centrifuged and stored at −80°C for future blood biochemistry analysis.

#### Echocardiography acquisition

Participants underwent a standardized 2-dimensional transthoracic echocardiographic scan using a commercially available Philips iE33 or EPIQ 7C cardiology ultrasound machine (Philips Healthcare Informatics). Image acquisition was made in the left lateral decubitus position per standard guidelines to include diastolic function measures (19). LV apical 4-, 3-, and 2-chamber views and short-axis views were acquired. Acquisition of pulsed-wave Doppler sample volumes between the mitral leaflet tips (E and A waves) was done using pulsed-wave Doppler with blood flow on apical 4-chamber views for later offline analysis of E/A ratio. Tissue Doppler imaging of the lateral and septal basal regions from apical 4-chamber views were also acquired for later offline analysis of average e′ velocity to calculate E/e′ ratio.

#### CMR acquisition

CMR was performed using a 3.0-T magnetic resonance imaging scanner (3T TIM Trio, Siemens Medical Solutions). Horizontal and vertical long-axis and LV outflow tract retrospective electrocardiographically gated steady-state free precession cine images were acquired, followed by short-axis steady-state free precession cine images (20). Data were acquired at end-expiratory breath-hold and digitally stored for offline analysis. LV short-axis images were obtained from the base to the apex of the heart. The thickness and gap between slices were 8.0 and 2.0 mm, respectively. A midventricular short-axis T1 map was acquired for each participant prior to LGE imaging (native T1 mapping) using the shortened modified Look-Locker inversion recovery sequence (21). LGE imaging was acquired using a T1-weighted phase-sensitive inversion recovery sequence in multiple short-axis slices to match cine views and long-axis planes. Images were acquired approximately 8 to 10 minutes after intravenous administration of a gadolinium-based contrast agent (Gadovist, Bayer) at a dose of 0.15 mmol/kg body weight. The inversion time was adjusted for optimal nulling of remote normal myocardium (22). Fifteen minutes after intravenous administration of gadolinium-based contrast agent, a midventricular short-axis T1 map (identical to the one acquired prior to LGE) was acquired.

### Image analysis

#### Assessment of function by echocardiography

Echocardiographic analysis of LV E/A and E/e′ ratios was completed using Philips IntelliSpace Cardiovascular 2.1 (Philips Healthcare Informatics) in accordance with standard guidelines (23). The E and A waves were calculated from the pulsed-wave Doppler with blood flow as the peak modal velocities in early and late diastole, respectively, at the leading edge of the spectral waveform, while e′ was calculated from the tissue Doppler imaging as the peak modal velocity in early diastole at the leading edge of the spectral waveform. Echocardiographic myocardial deformation was measured using Image Arena 4.6 (TomTec). LV longitudinal strain parameters were measured from apical 4-, 2-, and 3-chamber views and LV circumferential strain parameters from the midventricular short-axis view.

#### LV volumes, mass, dimensions, and function by CMR

LV CMR analysis was done using analytical software (CVI42, Circle Cardiovascular Imaging). To evaluate LV volumes, mass, and dimensions, both the epicardial and endocardial borders were manually contoured on short-axis cine images for each slice at end-diastole and endocardial borders at end-systole. The end-diastolic and end-systolic cardiac phases, as well as the basal and apical LV slices, were visually determined as previously described (20,24). LV wall thickness was measured on the midventricular short-axis slice at end-diastole. Myocardial deformation analysis was performed using feature tracking (CVI42). Endocardial and epicardial borders of the LV long-axis cines and midventricular short-axis cine were manually contoured on the end-diastolic frame. The deformation of the myocardium was then automatically tracked through the cardiac cycle to calculate systolic and diastolic longitudinal and circumferential strain parameters.

#### LV tissue characterization by CMR

Quantitative analysis of LGE and T1 maps was done using commercially available software (CVI42). For LGE analysis, the endocardial and epicardial myocardial borders at end-diastole were contoured for all short-axis slices. To quantify LGE mass, a signal intensity threshold of 5 SDs above the mean intensity of a reference region of interest was placed in a remote area of myocardium with no visual evidence of enhancement. The relative LGE mass was calculated as the percentage of total LV myocardial mass (LGE mass/LV mass × 100%). For T1 mapping analysis, myocardial contours were drawn on native T1 images and postcontrast T1 images. Blinded and experienced analysts undertook all analyses. Native and postcontrast average myocardial T1 values were generated for each participant. R2 maps of shortened modified Look-Locker inversion recovery fit were used to verify the quality of T1 maps. To allow the calculation of ECV, a measure of diffuse myocardial fibrosis (16), native and postcontrast blood T1 values were also measured.

### Statistical analysis

Statistical analysis was performed using SPSS version 26 (IBM). Shapiro-Wilk testing and visual inspection were used to assess normality of variables. Comparisons between preterm-born and term-born adults were performed using independent-samples Student’s *t*-tests for normally distributed data and Mann-Whitney and Kruskal-Wallis tests for skewed data. Group comparisons between the preterm-born and term-born adults were adjusted for sex to account for differing sex distributions between groups using multivariable linear regression, with adjusted mean differences and 95% confidence intervals (CIs) reported for the variables of interest. Bivariate and multivariable linear regression modeling were completed using forced entry with missing data excluded pairwise. For each analysis, unstandardized coefficients (*B*) with 95% CI, standardized coefficients (β), and *P* values are reported. Mediation analyses were performed to determine potential mediating pathways among gestational age, ECV, and diastolic function parameters after first performing bivariate regressions among these parameters (25). To determine if the mediation effect was significant, bootstrapping was used, and the indirect effect with 95% CI was computed (26). Statistical significance was determined at *P* < 0.05. *P* values and 95% CIs presented have not been adjusted for multiplicity, and therefore inferences drawn from these statistics may not be reproducible.

## Results

### Participant characteristics

A total of 149 participants were recruited into YACHT. For this study, 48 of the 149 participants from YACHT were excluded from analysis to prevent the potential confounding effects of hypertensive cardiac remodeling on our results (n = 32 from the hypertension clinic being treated pharmacologically and n = 16 with high blood pressure from 24-hour blood pressure monitoring). Of the 101 normotensive young adult participants included in this analysis, 54 were born at term (39.5 ± 1.4 gestational weeks), and 47 were born preterm (32.8 ± 3.2 gestational weeks). The cohort size allowed us to detect a 0.65-SD difference between groups powered at 90% and *P* = 0.05. There were no differences in age and body mass index between preterm-born and term-born groups (*P* > 0.05). The percentage of men was lower in the group of young adults born preterm compared with the term-born group, and as such, group comparisons throughout were adjusted for sex. All biochemistry measures, except for high-density lipoprotein and C-reactive protein, differed between groups (*P* < 0.05). There were no differences in average awake ambulatory systolic (119.2 ± 6.3 mm Hg vs 119.0 ± 7.6 mm Hg; adjusted mean difference 0.4 mm Hg; *P* = 0.610) and diastolic blood pressure between groups (70.8 ± 4.9 mm Hg vs 69.4 ± 4.5 mm Hg; adjusted mean difference 1.6 mm Hg; *P* = 0.058) (Table 1).

**Table 1:** Cohort Characteristics

	Preterm-Born Adults (n = 47)	Term-Born Adults (n = 54)	*P* Value
Demographics and anthropometrics			
Age, y	22.7 ± 3.0	23.6 ± 3.8	0.239
Male	14 (30.0)	26 (48.0)	0.061
Height, cm	167 ± 8.6	175 ± 10.0	**<0.001**
Weight, kg	65.3 ± 13.5	70.0 ± 13.0	0.299
BMI, kg/m^2^	23.3 ± 4.5	22.7 ± 2.7	0.436
Gestational age, wk	32.8 ± 3.2	39.5 ± 1.4	**<0.001**
32-36 wk	38 (80.9)	—	—
28-31 wk	5 (10.6)	—	—
<28 wk	4 (8.5)	—	—
Birth weight, g	1,916 ± 806	3,390 ± 424	**<0.001**
Birth weight range, g	595-3,203	2,640-4,536	**<0.001**
Gestational hypertension	8 (17.0)	0 (0.0)	**0.002**
Small for gestational age	2 (4.3)	0 (0.0)	0.214
Biochemistry			
Total cholesterol, mmol/L	4.72 ± 0.65	4.18 ± 0.77	**0.001**
HDL, mmol/L	1.49 ± 0.31	1.47 ± 0.26	0.953
LDL, mmol/L	2.80 ± 0.71	2.32 ± 0.60	**0.001**
Triglycerides, mmol/L	1.12 ± 0.66	0.87 ± 0.36	**0.031**
High-sensitivity CRP, mg/L	1.57 ± 2.42	1.14 ± 1.96	0.412
Glucose, mmol/L	5.02 ± 0.41	4.82 ± 0.51	**0.030**
Insulin, pmol/L	51.1 ± 29.0	35.8 ± 29.4	**0.012**
Insulin resistance	0.96 ± 0.54	0.68 ± 0.59	**0.020**
Brachial blood pressure			
Awake ambulatory systolic, mm Hg	119 ± 6	119 ± 8	0.610
Awake ambulatory diastolic, mm Hg	71 ± 5	69 ± 5	0.058

Values are mean ± SD, n (%), or range. *P* values are adjusted for sex. *P* values in **bold** indicate statistical signiﬁcance (*P* < 0.05).

BMI = body mass index; CRP = C-reactive protein; HDL = high-density lipoprotein; LDL = low-density lipoprotein.

### Altered cardiac structure, lower function, and greater diffuse myocardial fibrosis in adults born preterm

CMR showed that preterm-born adults had smaller LV volumes indexed to body surface area compared with term-born adults, including end-diastolic volume (72.02 ± 10.81 mL/m^2^ vs 88.21 ± 11.49 mL/m^2^; adjusted mean difference −14.56 mL/m^2^; *P* < 0.001), end-systolic volume (27.81 ± 8.90 mL/m^2^ vs 33.40 ± 6.61 mL/m^2^; adjusted mean difference −4.93 mL/m^2^; *P* < 0.001), and stroke volume (44.20 ± 7.50 mL/m^2^ vs 54.81 ± 7.32 mL/m^2^; adjusted mean difference −9.63 mL/m^2^; *P* < 0.001). Preterm-born adults also had greater LV average wall thickness (8.33 ± 0.84 mm vs 5.80 ± 0.96 mm; adjusted mean difference 2.68 mm; *P* < 0.001), mass (104.77 ± 19.31 g vs 98.06 ± 18.32 g; adjusted mean difference 11.28 g; *P* < 0.001), mass index (60.01 ± 7.25 g/m^2^ vs 53.05 ± 6.80 g/m^2^; adjusted mean difference 8.29 g/m^2^; *P* < 0.001), and mass/end-diastolic volume ratio (0.87 ± 0.09 g/mL vs 0.60 ± 0.10 g/mL; adjusted mean difference 0.26 g/mL; *P* < 0.001) compared with term-born adults (Table 2).

**Table 2:** Left Ventricular Structure, Function, and Myocardial Tissue Characterization

	Preterm-Born Adults (n = 47)	Term-Born Adults (n = 54)	*P* Value
CMR structural parameters			
End-diastolic volume/BSA, mL/m^2^	72.02 ± 10.81	88.21 ± 11.49	**<0.001**
End-systolic volume/BSA, mL/m^2^	27.81 ± 8.90	33.40 ± 6.61	**<0.001**
Myocardium mass, g	104.77 ± 19.31	98.06 ± 18.32	**<0.001**
Myocardium mass/BSA, g/m^2^	60.01 ± 7.25	53.05 ± 6.80	**<0.001**
Mass/end-diastolic volume, g/mL	0.87 ± 0.09	0.60 ± 0.10	**<0.001**
Average wall thickness, mm	8.33 ± 0.84	5.80 ± 0.96	**<0.001**
CMR functional parameters			
Ejection fraction, %	61.33 ± 4.44	62.28 ± 4.52	0.325
Stroke volume/BSA, mL/m^2^	44.20 ± 7.50	54.81 ± 7.32	**<0.001**
Longitudinal systolic strain, %	−15.35 ± 1.58	−18.75 ± 2.27	**<0.001**
Longitudinal systolic strain rate, s^−1^	−0.93 ± 0.20	−1.08 ± 0.24	**0.002**
Circumferential systolic strain, %	−18.31 ± 2.09	−18.48 ± 2.24	0.893
Circumferential systolic strain rate, s^−1^	−1.03 ± 0.14	−1.03 ± 0.21	0.953
Longitudinal diastolic strain rate, s^−1^	0.99 ± 0.23	1.14 ± 0.26	**0.009**
Circumferential diastolic strain rate, s^−1^	1.54 ± 0.38	1.65 ± 0.34	0.203
Echocardiographic functional parameters			
Longitudinal systolic strain, %	−19.32 ± 2.72	−20.15 ± 3.16	**0.019**
Longitudinal systolic strain rate, s^−1^	−1.04 ± 0.18	−1.18 ± 0.31	0.063
Circumferential systolic strain, %	−25.66 ± 4.01	−26.70 ± 3.22	0.321
Circumferential systolic strain rate, s^−1^	−1.67 ± 0.30	−1.57 ± 0.28	0.156
Longitudinal diastolic strain rate, s^−1^	1.49 ± 0.39	1.82 ± 0.61	**0.008**
Circumferential diastolic strain rate, s^−1^	2.20 ± 0.70	2.26 ± 0.58	0.706
+E/A ratio	1.52 ± 0.38	1.75 ± 0.42	**0.013**
+E/e’ ratio	4.90 ± 1.13	4.96 ± 1.04	0.768
CMR myocardial tissue characterization			
ECV, %	27.81 ± 1.69	25.48 ± 1.41	**<0.001**
Native T1 myocardium, ms	1173.81 ± 29.29	1169.24 ± 41.13	0.908
LGE mass, g	0.45 (0.049-3.04)	0.43 (0.027-3.51)	0.845
Relative LGE mass, %	0.42 (0.034-3.81)	0.44 (0.035-3.85)	0.557

Values are mean ± SD or median (range). *P* values are adjusted for sex. *P* values in **bold** indicate statistical signiﬁcance (*P* < 0.05). Average wall thickness and circumferential strain measures are from the midventricular short-axis slice.

BSA = body surface area; CMR = cardiovascular magnetic resonance; ECV = extracellular volume; LGE = late gadolinium enhancement.

There were no differences between preterm-born and term-born adults for LV ejection fraction (*P* = 0.325) or midventricular peak systolic circumferential strain (*P* = 0.893) and strain rate (*P* = 0.953), though both longitudinal peak systolic strain (−15.35% ± 1.58% vs −18.75% ± 2.27%; adjusted mean difference 3.31%; *P* < 0.001) and strain rate (−0.93 ± 0.20 s^–1^ vs −1.08 ± 0.24 s^−1^; adjusted mean difference 0.16 s^−1^; *P* = 0.002) were lower in those born preterm. Although midventricular peak diastolic strain rate did not differ between preterm-born and term-born adults (*P* = 0.203), longitudinal peak diastolic strain rate was lower in the preterm group (0.99 ± 0.23 s^–1^ vs 1.14 ± 0.26 s^−1^; adjusted mean difference −0.14 s^−1^; *P* = 0.009). On echocardiography, there was no difference in E/e′ ratio (*P* = 0.768), but E/A ratio was lower in preterm-born adults compared with term-born adults (1.52 ± 0.38 vs 1.75 ± 0.42; adjusted mean difference −0.22; *P* = 0.013).

**Figure 1:**
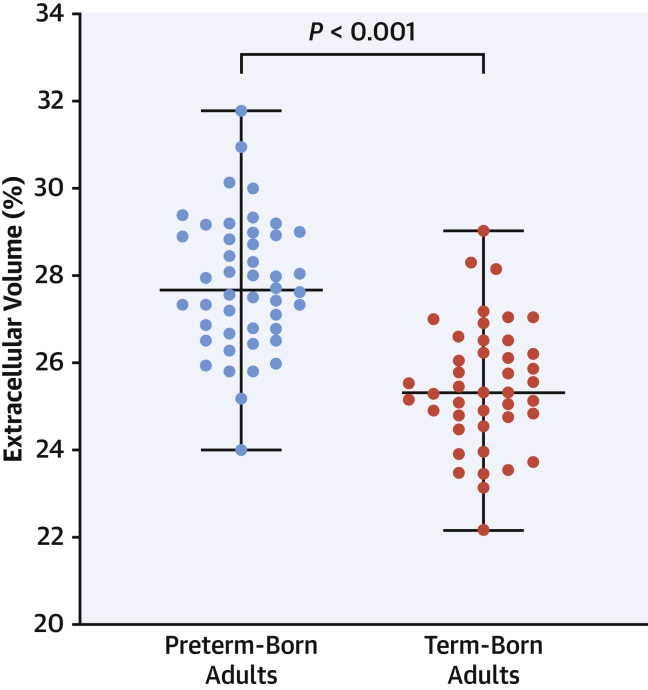
Greater Extracellular Volume Fraction in Adults Born Preterm Preterm-born adults **(blue)** had higher extracellular matrix volume compared with term-born adults **(red)** (27.81% ± 1.69% vs 25.48% ± 1.41%; *P* < 0.001). Group comparisons were adjusted for sex (adjusted mean difference 2.17%; 95% confidence interval: 1.46-2.87). Box-and-whisker plots presented as median and range.

Although native T1 mapping did not differ between groups (*P* = 0.908), ECV was higher in the preterm-born adults compared with term-born adults (27.81% ± 1.69% vs 25.48% ± 1.41%; adjusted mean difference 2.17%; *P* < 0.001) with 4 of 47 adults born preterm (9%) having ECV >3 SDs (∼30%) above the mean defined by our term-born adult group (Figure 1). Furthermore, 9 of 47 of the preterm-born adults (19%) had ECV measures above the upper limit of the term-born adult comparison group. Differences could not be explained by alterations in hematocrit, as there were no differences between the preterm-born and term-born adults (0.43 ± 0.02 L/L vs 0.44 ± 0.03 L/L; adjusted mean difference −0.005 L/L; *P* = 0.222). LGE mass and relative LGE mass did not differ between groups (*P* = 0.845 and *P* = 0.557, respectively).

### Diffuse fibrosis mediates the relationship between the degree of prematurity and diastolic function

LV cardiac structure and function parameters that differed in those born preterm compared with term were selected for bivariate regressions with ECV (Table 3). Longitudinal peak diastolic strain rate (CMR: β = −0.454; *P* = 0.010; echocardiography: β = −0.395; *P* = 0.027) and E/A ratio (β = −0.464; *P* = 0.011) were the only parameters inversely related to ECV. In bivariate regression analysis within the preterm group, gestational age was inversely related with ECV, with a 0.289% lower ECV (*P* = 0.001) per 1-week greater gestational age. Gestational age was also positively related with E/A ratio and longitudinal peak diastolic strain rate, with a 0.041-unit higher E/A ratio (*P* = 0.020), 0.075-s^−1^ higher CMR-derived longitudinal peak diastolic strain rate (*P* = 0.016), and 0.083-s^−1^ higher echocardiography-derived longitudinal peak diastolic strain rate (*P* = 0.018) per 1-week greater gestational age.

**Table 3:** Relationship Between Left Ventricular Structural and Functional Changes and ECV in Preterm-Born Young Adults Using Bivariate Regression

	ECV, %				
	*B*	β	95% Confidence Interval for *B*		*P* Value
			Lower Bound	Upper Bound	
CMR myocardium mass/BSA, g/m^2^	−0.808	−0.188	−2.379	0.764	0.302
CMR mass/end diastolic volume, g/mL	0.006	0.109	−0.014	0.026	0.552
CMR average wall thickness, mm	−0.035	−0.122	−0.313	0.431	0.432
CMR stroke volume/BSA, mL/m^2^	−0.511	−0.115	−2.157	1.135	0.531
CMR longitudinal systolic strain, %	0.145	0.209	−0.113	0.403	0.260
CMR longitudinal systolic strain rate, s^−1^	−0.017	−0.099	−0.099	0.066	0.682
CMR longitudinal diastolic strain rate, s^−1^	**−0.386**	**−0.454**	**−0.673**	**−0.099**	**0.010**
Echocardiography longitudinal systolic strain, s^−1^	0.100	0.135	−0.221	0.421	0.433
Echocardiography longitudinal diastolic strain rate, s^−1^	**−0.293**	**−0.395**	**−0.510**	**−0.076**	**0.027**
Echocardiography E/A ratio	**−0.105**	**−0.464**	**−0.185**	**−0.026**	**0.011**

Values in **bold** are statistically significant (*P* < 0.05). *B* represents the difference in left ventricular structural or functional variables per 1% elevation in ECV (slope or unstandardized regression coefficient). β represents the standardized regression coefficient (−1 to 1).

Abbreviations as in Table 2.

**Figure 2:**
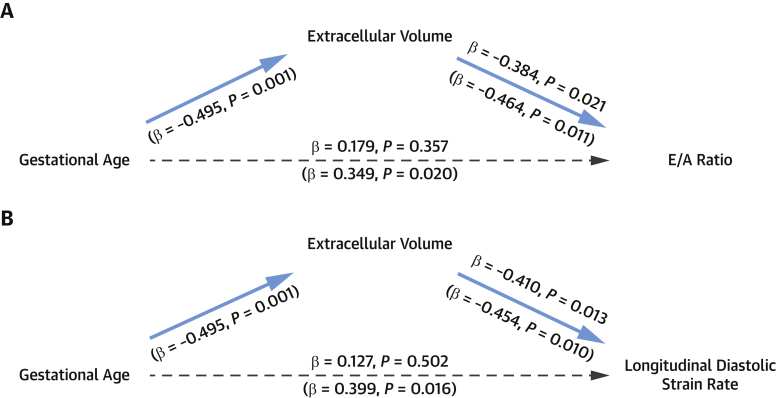
ECV Mediates the Relationship Between Gestational Age and Diastolic Function **(A)** Gestational age was significantly related to extracellular volume fraction (ECV) (β = −0.495; *P* = 0.001) and E/A ratio (β = 0.349; *P* = 0.020) (shown in **parentheses below the arrows**). When controlling for ECV, gestational age was no longer a significant predictor of E/A ratio (β = 0.179; *P* = 0.357) (shown above the **arrows**). **(B)** Gestational age was significantly related to ECV (β = −0.495; *P* = 0.001) and cardiovascular magnetic resonance longitudinal peak diastolic strain rate (β = 0.399; *P* = 0.016) (shown in **parentheses below the arrows**). When controlling for ECV, gestational age was no longer a significant predictor of longitudinal peak diastolic strain rate (β = 0.127; *P* = 0.502) (shown **above the arrows**).

Mediation analyses were performed to determine whether the relationships between the degree of prematurity and impaired diastolic function were mediated by diffuse myocardial fibrosis (ECV). Controlling for gestational age did not alter the association between ECV and E/A ratio (β = −0.384; *P* = 0.021), but gestational age was no longer a significant predictor of E/A ratio (β = 0.179; *P* = 0.357) (Figure 2A). Controlling for gestational age also did not alter the association between ECV and CMR-derived longitudinal peak diastolic strain rate (β = −0.410; *P* = 0.013), but gestational age was no longer a significant predictor of CMR-derived longitudinal peak diastolic strain rate (β = 0.127; *P* = 0.502) (Figure 2B). Similarly, controlling for gestational age did not alter the association between ECV and echocardiography-derived longitudinal peak diastolic strain rate (β = −0.283; *P* = 0.044), but gestational age was no longer a significant predictor of echocardiography-derived longitudinal peak diastolic strain rate (β = 0.205; *P* = 0.183). The indirect effect of gestational age on E/A ratio via ECV was significant (mediation effect 0.0063; 95% CI: 0.0019-0.0133), as was the indirect effect of gestational age on CMR-derived longitudinal peak diastolic strain rate via ECV (mediation effect 0.0043; 95% CI: 0.0009-0.0084) and echocardiography-derived longitudinal peak diastolic strain rate via ECV (mediation effect 0.0055; 95% CI: 0.0012-0.0098).

## Discussion

This study demonstrates for the first time that young adults born preterm have evidence of greater diffuse myocardial fibrosis in the LV that relates to the degree of prematurity (Central Illustration). Preterm-born adults also exhibit LV structural and functional changes, including smaller LV end-diastolic and stroke volumes, greater mass, and lower systolic and diastolic function. Impairments in diastolic function by both CMR and echocardiography relate to the greater diffuse myocardial fibrosis as well as the degree of prematurity. These associations between gestational age and reductions in diastolic function are mediated by the greater diffuse myocardial fibrosis present in the LV of preterm-born adults.

**Central Illustration:**
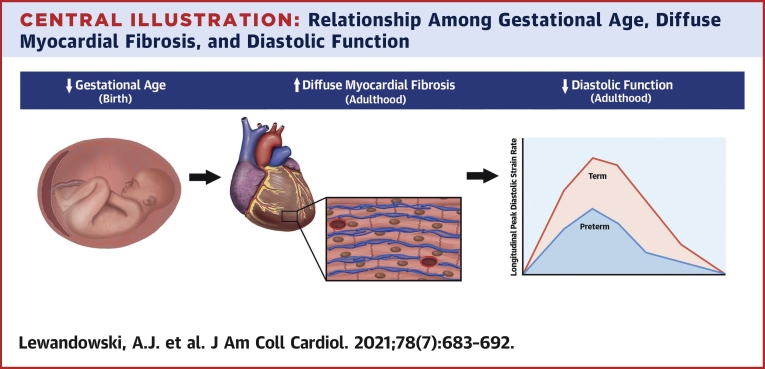
Relationship Among Gestational Age, Diffuse Myocardial Fibrosis, and Diastolic Function Compared with their term-born peers (n = 54), preterm-born young adults (n = 47) had higher left ventricular extracellular volume fraction, which is a surrogate measure of diffuse myocardial fibrosis measured by cardiovascular magnetic resonance. They were also shown to have lower left ventricular diastolic function in young adulthood measured by both cardiovascular magnetic resonance and echocardiography. A greater degree of prematurity (lower gestational age) associated with elevated diffuse myocardial fibrosis and impaired diastolic function, with diffuse myocardial fibrosis shown to be a significant mediator in the association between gestational age and diastolic function. Further follow-up will be needed to determine the clinical significance of these left ventricular changes in structure and function in people born preterm as they reach middle to late adulthood.

ECV has emerged as a sensitive surrogate marker for interstitial myocardial fibrosis (16). Unlike LGE, which reflects replacement or focal fibrosis, ECV captures reactive fibrosis and better reflects early stages of cardiac remodeling (27). Comparison studies have confirmed that CMR ECV measures correlate strongly with histologic collagen volume fraction (28,29) and demonstrate a better agreement with histologic measurements than native T1 mapping (30). The absence of any difference between preterm-born and term-born groups for LGE and the presence of a significant difference for ECV suggests that there may be greater reactive fibrosis, rather than replacement fibrosis, in the myocardial wall of preterm-born young adults. Although the predictive value of ECV for later cardiovascular events has not been studied in young adult populations, it has been shown to be significantly associated with future heart failure events, independent of replacement fibrosis, in a large middle-aged cohort. Indeed, elevated ECV levels >30% were shown to relate to an independent, 1.82-fold increased risk for death and 1.60-fold increased risk for heart failure hospitalization. As such, ECV is now recognized as a novel prognostic marker independent of noninfarction scar in the myocardium (27). In our present study of normotensive individuals, we identified that nearly 9% of the preterm population had ECV of 30% or greater, which may contribute to their greater risk for early heart failure (4) and early cardiovascular-related mortality (7). Our finding of greater ECV with no change in native T1 in the preterm-born adults suggests a specific association with collagen deposition. T1 can vary with several biological factors, whereas ECV is considered to more specifically reflect alterations in collagen deposition (16). Nevertheless, it is possible that ECV may be elevated because of other unknown factors.

The sustained alterations in cardiac morphology and performance exhibited by people born preterm (8) may be due in part to the hemodynamic stress and pressure overload faced by the immature myocardium around the time of birth as it transitions to the high-resistance extrauterine environment (31). These changes in flow through the cardiac chambers and higher magnitude of shear stress on the myocardial wall are common triggers for reactive fibrosis, characterized by excessive deposition within the extracellular matrix (32). Cardiac statistical atlases created from CMR images have revealed that the preterm young adult heart has an abnormal LV and right ventricular geometry (20,24), with smaller internal cavity dimensions and shorter ventricular lengths. Similar dimensional reductions to those in preterm-born young adults have been observed in neonates, infants, children, and adolescents born preterm (8,9). This more globular cardiac structure could also alter flow dynamics through the cardiac chambers, leading to a consistent elevation in myocardial wall shear stress that continuously drives progression of reactive fibrosis to maintain cardiac output. Further studies are needed to assess whether cardiac morphology in people born preterm affects intraventricular wall shear stress and flow dynamics (33).

Additional stimuli, including inflammation and oxidative stress, are also likely to be involved in the pathogenesis of extracellular matrix fibrosis in people born preterm (34). In humans, preterm neonates and infants exhibit acute hyperinflammatory states with a reduced antioxidant capacity, which often fail to resolve (35) and are common triggers of fibrosis (36). In rats, hyperoxia exposure to mimic preterm birth–related conditions stimulates activation of Toll-like receptor 4 in the left ventricle (34), which contributes to cardiac remodeling and dysfunction by promoting myocardial inflammation, oxidative stress, and fibrosis. Transient treatment of rats during the neonatal period with a Toll-like receptor 4 antagonist in this preterm model prevents negative long-term cardiac remodeling, including the greater interstitial myocardial fibrosis and impaired diastolic function (34). Overactivation of the renin-angiotensin system has been shown in the rat model of hyperoxia exposure, whereby neonatal treatment with an angiotensin II type 1 receptor blocker prevents the development of myocardial fibrosis in adulthood (37). Targeting Toll-like receptor 4 inflammatory pathways and renin-angiotensin system signaling to reduce myocardial fibrosis and abnormal cardiac remodeling following preterm birth warrants further investigation in humans.

An important negative finding within the preterm-born adults was the lack of an association between higher LV mass and wall thickness with greater ECV. This supports the hypothesis that the higher myocardial mass following preterm birth is driven by cardiomyocyte hypertrophic growth through other mechanisms, such as the adaptation to early hemodynamic changes faced by the immature myocardium (3,11). However, there remains discrepancy between preterm-born adult cohorts, with some demonstrating lower LV mass (9), which may relate to reduced cardiac endowment with earlier gestations or differences in clinical care across health care systems (10). We found a strong relationship between ECV and impairments in both CMR- and echocardiography-derived diastolic function measures in the preterm group. The expansion of the extracellular matrix through up-regulation of fibrotic pathways can have a significant impact on cardiac function and in particular may lead to diastolic dysfunction as a result of mechanical stiffness (38). Furthermore, excessive reactive fibrosis can directly impede systolic function. Although we did not identify any associations with systolic strain parameters, it is possible that as these young adults age, and diffuse myocardial fibrosis continues to increase (27), systolic function will be further affected. Although E/A ratio and longitudinal peak diastolic strain rate were reduced in preterm-born adults, E/e′ ratio was similar between groups, though this may also be impaired in cohorts of preterm-born individuals with lower gestational ages (2). Follow-up of our cohort to determine if elevated ECV relates to impairments in E/e′ ratio over time will be of interest.

### Study limitations

First, despite the associations among gestational age, ECV, and diastolic function, further work is needed to explore this relationship across gestational age ranges, given the small number of individuals in the very and extreme preterm group (<32 gestational weeks) in our cohort.

Second, given the cross-sectional nature of the study, we cannot confirm causality and whether changes in ECV occurred around the time of birth or have emerged over time. Nevertheless, our analyses showing that ECV is a mediator in the relationship between the degree of prematurity and diastolic functional impairments, which are first seen during infancy (39), suggests that early life events are important to the presence of ECV in young adulthood.

Third, our measure of ECV is based on a single, midventricular short-axis slice. However, slice position was standardized on the basis of the presence of papillary muscles, and our methodology is consistent with other studies that have shown ECV is a biopsy-validated surrogate measure of interstitial fibrosis (16,29). Furthermore, our results demonstrate consistent relationships with diastolic functional impairments, measured by both CMR and echocardiography.

Finally, previous studies have demonstrated that the right ventricle has greater functional impairments than the left ventricle in young adults born preterm (40), independent of changes in pulmonary physiology (24). It is possible that diffuse myocardial fibrosis has a greater impact on the thinner walled right ventricle, leading to an accelerated reduction in function. However, we have not quantified this in our study because of the technical limitations of analyzing ECV for the right ventricle (41).

## Conclusions

We have demonstrated that preterm-born young adults have alterations in LV morphology and function, including greater diffuse myocardial fibrosis that mediates the relationship between the degree of prematurity and impaired diastolic function (Central Illustration). Greater diffuse myocardial fibrosis may underlie part of the increased cardiovascular risk in this population, including heart failure, ischemic heart disease, and early cardiovascular-related mortality.

Perspectives **COMPETENCY IN MEDICAL KNOWLEDGE:** Preterm birth is associated with an increased risk for cardiovascular diseases and diastolic ventricular dysfunction in young adulthood, possibly mediated by myocardial fibrosis.

**TRANSLATIONAL OUTLOOK:** Further research is needed to determine whether myocardial fibrosis in young adults born preterm worsens with aging and acquisition of conventional cardiovascular risk factors.

## Funding Support and Author Disclosures

This study was funded by a British Heart Foundation project grant (PG/13/58/30397). Dr Lewandowski is funded by a British Heart Foundation Intermediate Research Fellowship (FS/18/3/33292). The authors have reported that they have no relationships relevant to the contents of this paper to disclose.
